# Supplementary material for: Single‐Nanoparticle Detection Using Quasi‐Bound States in the Continuum Supported by Silicon Metasurfaces
Source: Small Methods. 2026 Aug 4;10(17):e70931. doi: 10.1002/smtd.70931 (PMC13555652; doi:10.1002/smtd.70931)
Supplement: Supplementary file 1 [file SMTD-10-e70931-s001.pdf]

Supporting Information for

Single-Nanoparticle Detection Using Quasi-Bound States in the Continuum Supported by Silicon Metasurfaces

Keisuke Watanabe[1]*, Samuel Crowther[2], Masanobu Iwanaga[3], Frank Vollmer[2], and Tadaaki Nagao[1,4]

1.* International Center for Materials Nanoarchitectonics (MANA), National Institute for Materials Science (NIMS), 1-1 Namiki, Tsukuba, Ibaraki 305-0044, Japan.
2. Department of Physics and Astronomy, Living Systems Institute, University of Exeter, EX4 4QD, Exeter, UK.
3. Research Center for Electronic and Optical Materials, National Institute for Materials Science (NIMS), 1-1 Namiki, Tsukuba, Ibaraki 305-0044, Japan.
4. Department of Condensed Matter Physics, Graduate School of Science, Hokkaido University, Kita 10, Nishi 8, Kita-ku, Sapporo 060-0810, Japan.

*Email: watanabe.keisuke@nims.go.jp

S1. Characterization of the laser beam diameter

We employed the knife-edge method to characterize the beam profile and estimate the diameter of the focused laser beam on a silicon surface. A 40 µW laser beam was focused onto the silicon surface, and a beveled thin blade was positioned near the focal spot. As shown in Figure S1a, the blade was translated across the focused beam in 5 µm increments while the transmitted optical power was monitored using a photoreceiver. The spatial beam profile was derived by calculating the gradient of the measured power profile with respect to the blade position, as shown in Figure S1b. The resulting power gradient was well fitted by a Gaussian function. The beam diameter was determined as the full width at the $1/e^2$ maximum, yielding a beam diameter of 28.4 µm at the sample plane.

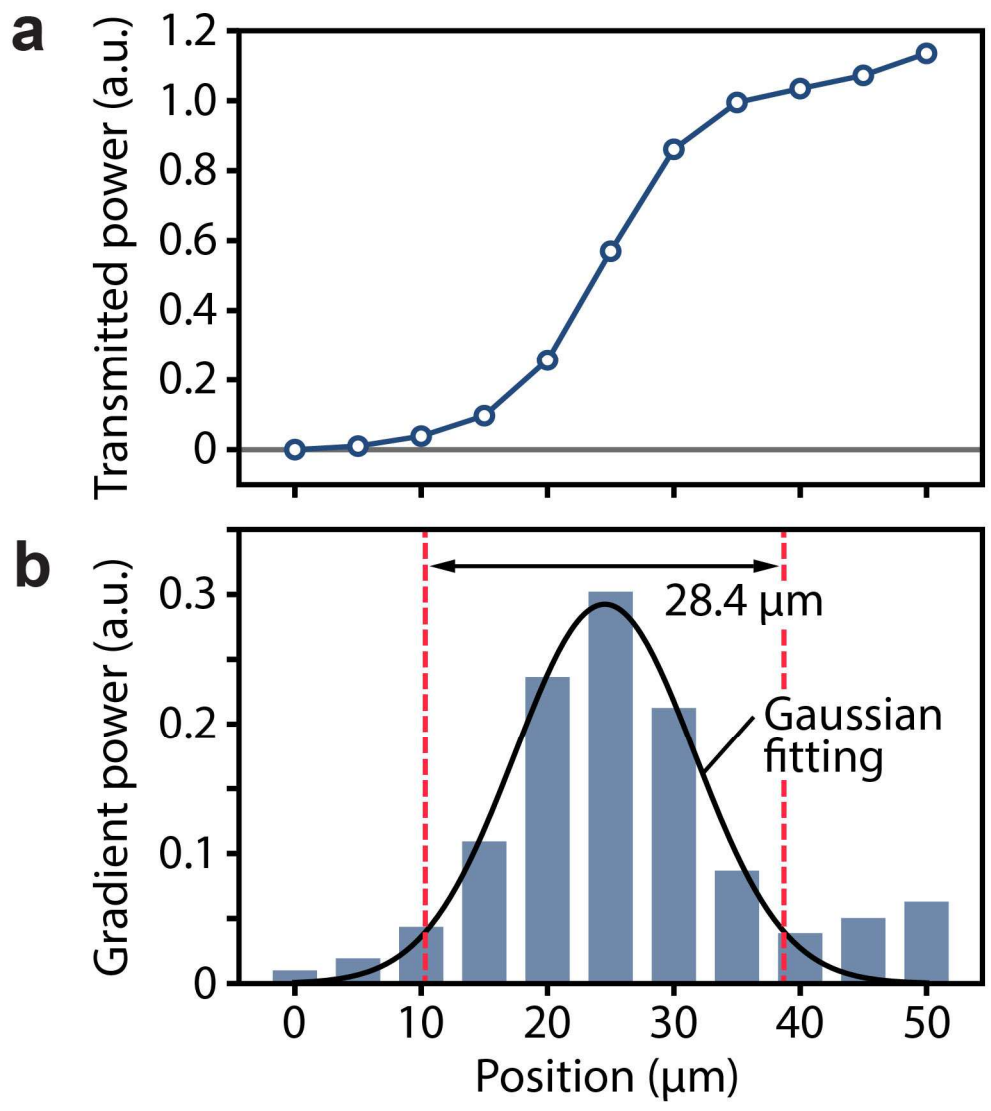

Figure S1. (a) Measured transmitted power as a function of knife-edge position. (b) Reconstructed beam profile together with the Gaussian fitting.

S2. *Q* factors considering water material absorption

To evaluate the influence of water absorption on the device performance, we performed additional FDTD simulations comparing the resonance *Q* factors in D_2O and H_2O. For H_2O, the imaginary part of the refractive index was incorporated using optical constants reported by Palik.[48] The results are summarized in the Figure S2 and Table S1. Considering that the resonance wavelengths of our qBICs are located near 1595 nm, we set the extinction coefficient *k* of water as $k = 0.0001$ (Figure S2a). We then calculated the *Q* factors for different asymmetry parameters α. The simulated results show that the *Q* factors in H_2O are only slightly lower than those in D_2O, particularly for small asymmetry parameters. Using the relation $Q^{-1} = Q_r^{-1} + Q_{abs}^{-1}$, absorption-limited *Q* factor associated with water was estimated to be approximately $Q_{abs} \approx 3.00 \times 10^8$. This value is several orders of magnitude larger than the radiative *Q* factors Q_r, indicating that water absorption contributes negligibly to the overall loss at the operating wavelength.

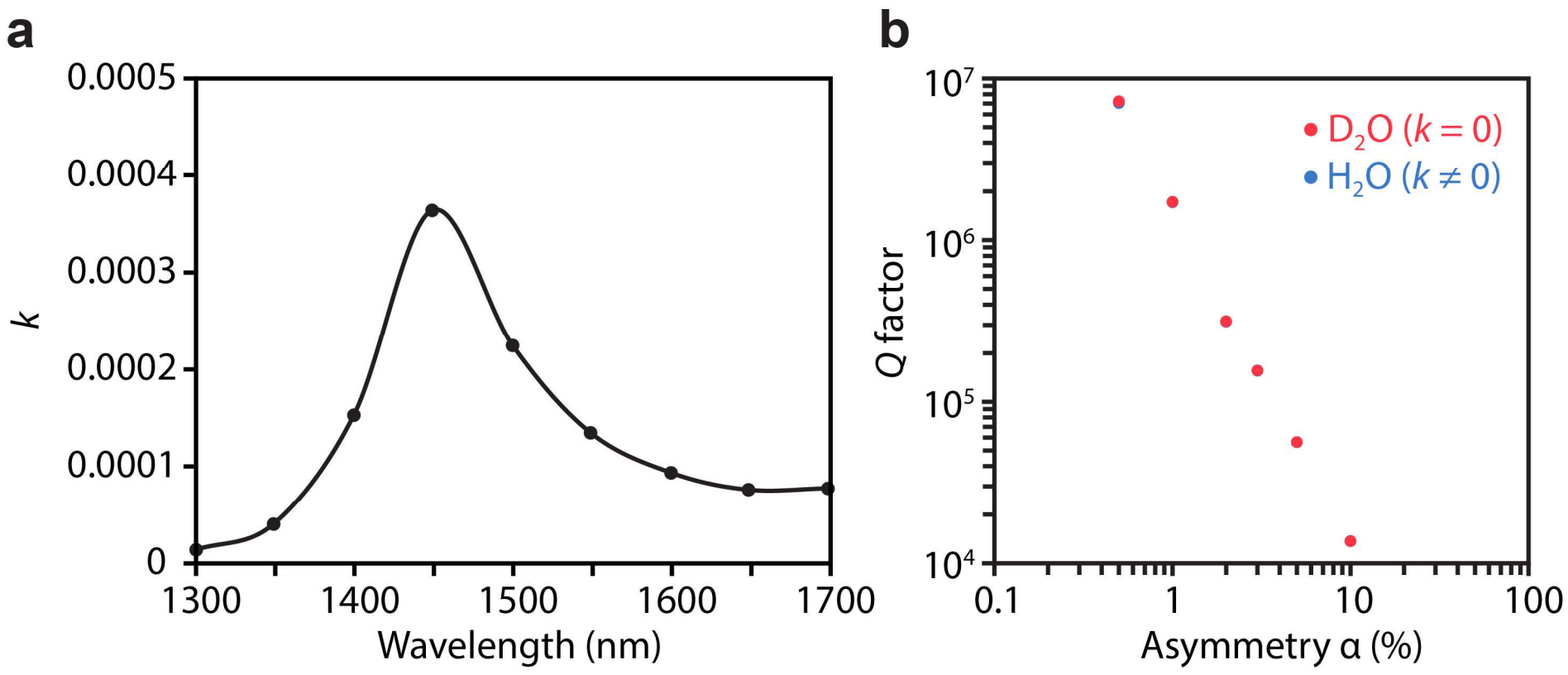

Figure S2. (a) Extinction coefficient *k* of water taken from Palik.[48] (b) Comparison of the FDTD calculated *Q* factors with (blue circles) and without (red circles) consideration of water absorption.

Table S1. Comparison of the FDTD calculated *Q* factors in H_2O and D_2O, together with the analytical values obtained from $Q^{-1} = Q_r^{-1} + Q_{abs}^{-1}$, for different asymmetry parameters.

**Asymmetry α (%)**	**0.5**	**1**	**2**	**3**	**5**	**10**	**12**
H_2O ($k \neq 0$)	7049960	1704370	314206	155340	56207.4	13707.3	9091.56
D_2O ($k = 0$)	7219160	1718420	315863	156040	56196.4	13671.5	9068.28
$(Q_r^{-1} + Q_{water}^{-1})^{-1}$	7049706	1708644	315531	155959	56185.9	13670.9	9068.01

S3. Transmission spectra of low-contrast BIC metasurfaces with different asymmetry parameters

Figure S3a shows the simulated transmittance map, where the refractive index of the surrounding medium was set to 1.33. The resonance amplitude and linewidth increase with an increase in the asymmetry parameter α, while the resonance wavelengths remain constant. A metasurface array with different α values was integrated into a PDMS microfluidic channel (Figure S3b), and the transmission spectra were measured in D_2O (Figure S3c). For α ≤ 3%, the laser output power was maintained at 50 μW. In this case, the resonance amplitude increased with increasing α. For α ≥ 4%, the laser output power was reduced to avoid detector saturation caused by the increased resonance amplitude, which confirmed that the amplitude continued to increase with α. Meanwhile, the resonance linewidth increased with α. These experimental observations were in good agreement with the simulated transmission spectra. Although slight fluctuations in the experimental resonance wavelength were observed for different α, the overall wavelength positions agreed well with the simulations.

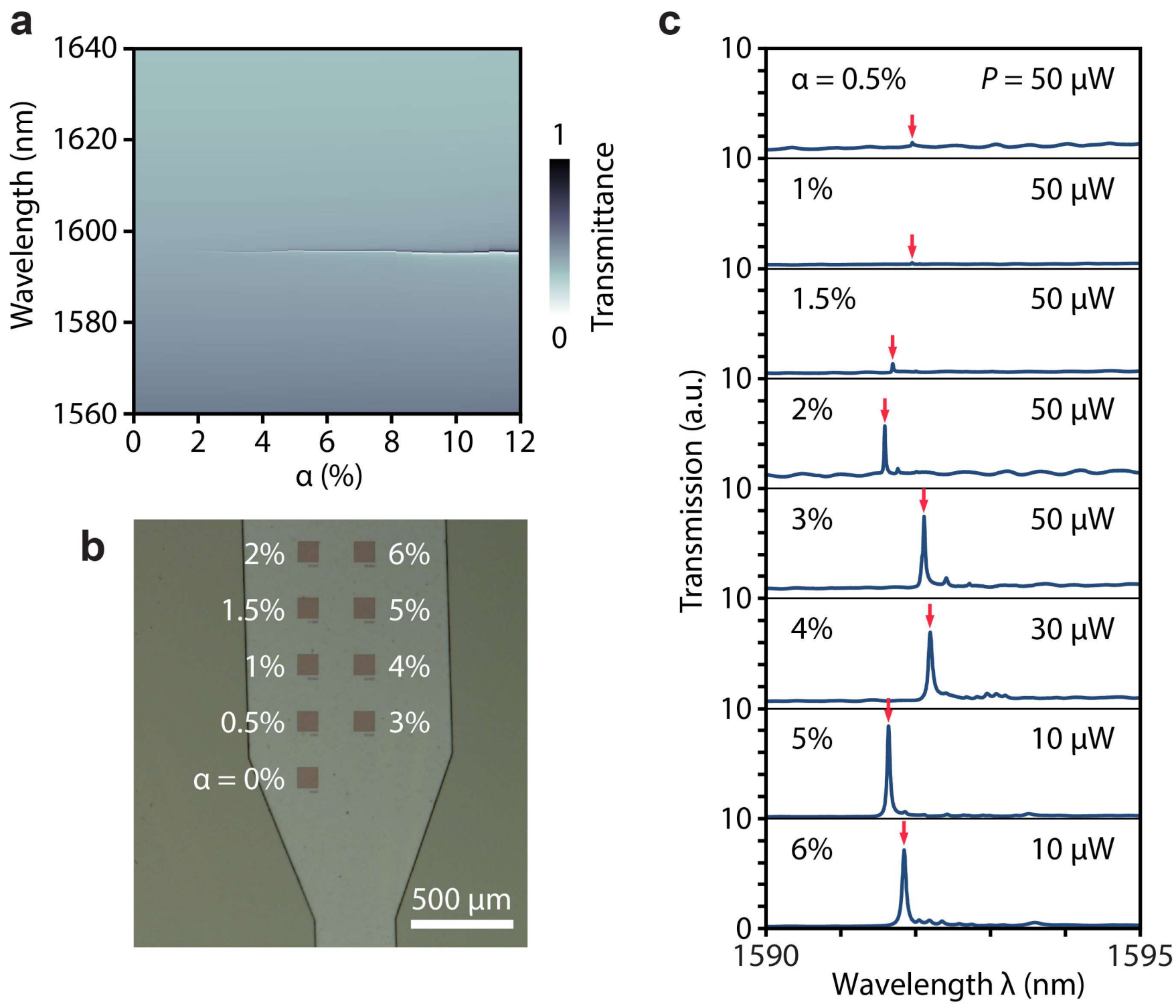

Figure S3. (a) Simulated transmittance map as a function of the asymmetry parameter α. (b) Optical microscope image of a fabricated device integrated with a PDMS microfluidic channel, showing a metasurface (100 μm x 100 μm) array with varied α. (c) Experimental transmission spectra measured for different *α*.

S4. Electric field profiles along the *z*-direction

Figure S4 shows the simulated electric field enhancement in the *yz*-plane at $x = 0$ nm and *xy*-plane at different *z* positions. An *x*-polarized plane wave was normally incident on a metasurface, and the electric field distribution at the qBIC resonance wavelength was monitored. In the *yz*-plane, two antinodes appear along the *y*- and *z*- directions. The field profile of the *xy*-plane at the position of maximum electric field intensity along the *z*-direction (z_{inmax}) indicates a single antinode along the *x*-direction. These field characteristics confirm that the higher-order qBIC originates from a TE-like TE_{122} mode. The confined optical mode partially penetrates the surrounding water because of the low-contrast geometry and large refractive index contrast between silicon and water. This results in strong electric field enhancement at the edges of the shallow-etched rods ($z_{surface}$). At the top silicon surface (z_{top}), the field enhancement at the rod edges becomes weaker and decays exponentially along the positive *z*-direction (z_{500}). These results confirm why the strongest light–molecule interactions occur near the shallow-etched regions.

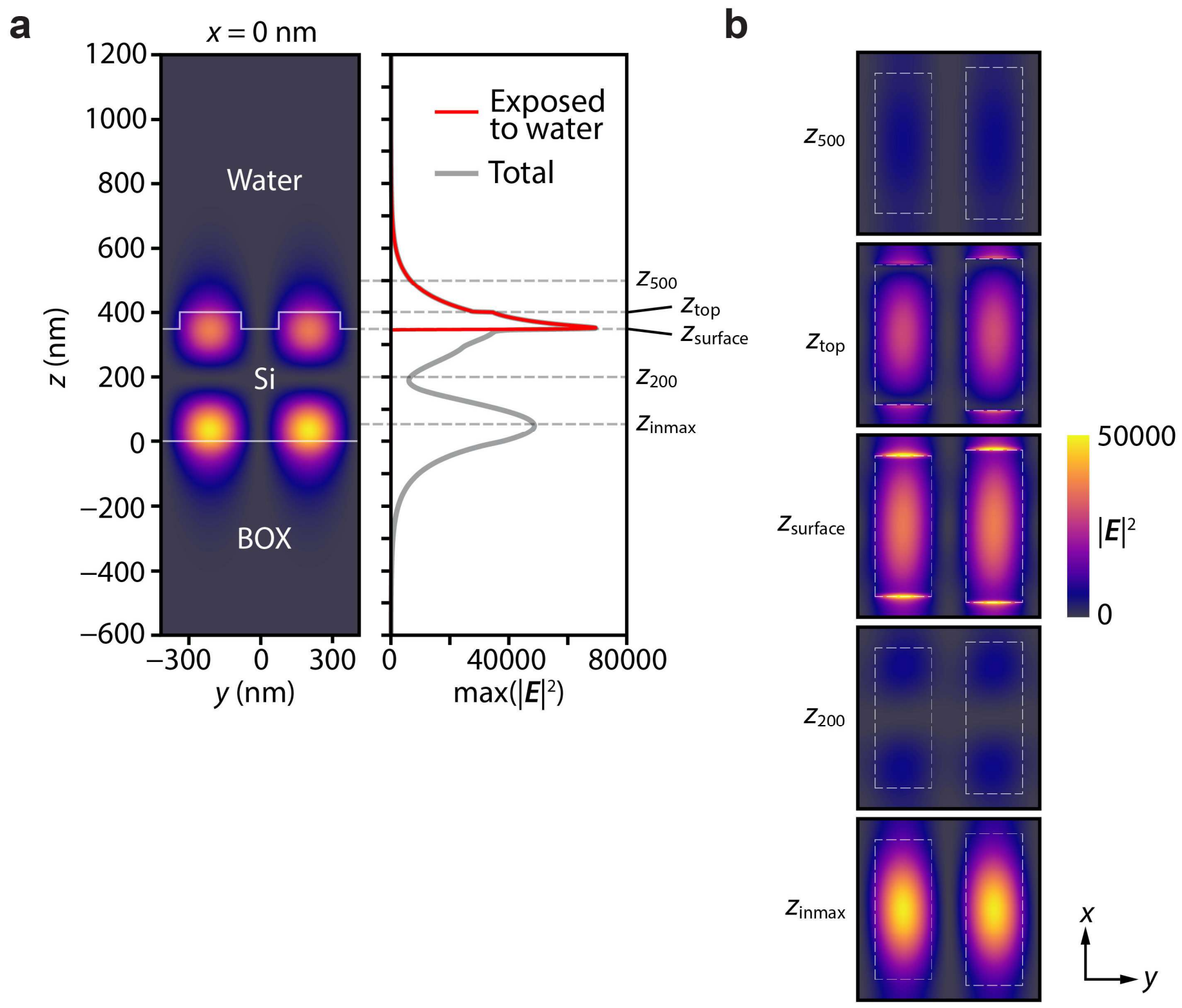

Figure S4. (a) Simulated electric field intensity $|E^2|$ at $x = 0$ nm in the *yz*-plane (left) and the corresponding maximum field intensity profile along the *z*-direction (right) for the metasurface with α = 4%. (b) Electric field distributions in the *xy*-plane at the different *z* positions (z_{500}, $z = 500$ nm; z_{top}, top silicon surface; $z_{surface}$, shallow-etched surface; z_{200}, $z = 200$ nm; z_{inmax}, position of maximum electric field intensity inside silicon).

S5. Statistical analysis of wavelength steps

Statistical significance of the wavelength step distributions was evaluated using Welch's ANOVA followed by Games–Howell multiple comparisons to account for unequal sample sizes and variances among particle-size groups. Welch's ANOVA revealed a significant effect of particle diameter on the wavelength step height ($F = 60.5$, $p < 0.001$). Post hoc analysis (Table S2) showed that the 100 nm particles produced significantly larger wavelength shifts than the 200 nm ($p < 0.001$), 500 nm ($p < 0.001$), and 1000 nm particles ($p = 0.039$). In addition, statistically significant differences were found between the 200 nm and 500 nm particle groups ($p = 0.0023$), and between the 200 nm and 1000 nm particle groups ($p = 0.0195$), but it was not the case between the 500 nm and 1000 nm particle groups ($p = 0.589$).

Table S2. Results of multiple-comparison tests for wavelength step heights obtained from different particle-size groups.

**Comparison (nm)**	**Mean difference (pm)**	**P value**	**Significant?**
100 vs. 200	2.35	<0.0001	✓
100 vs. 500	1.55	<0.0001	✓
100 vs. 1000	1.10	0.0388	✓
200 vs. 500	0.80	0.0023	✓
200 vs. 1000	1.25	0.0195	✓
500 vs. 1000	0.45	0.589	

S6. Statistical analysis of PS particle binding process

Figure S5 shows the histogram of the time intervals Δt between successive binding events for PS particles with d = 100 nm. The distribution follows a single-exponential decay, which is consistent with a Poissonian stochastic adsorption process. This statistical behavior indicates that individual binding events occur independently and probabilistically.

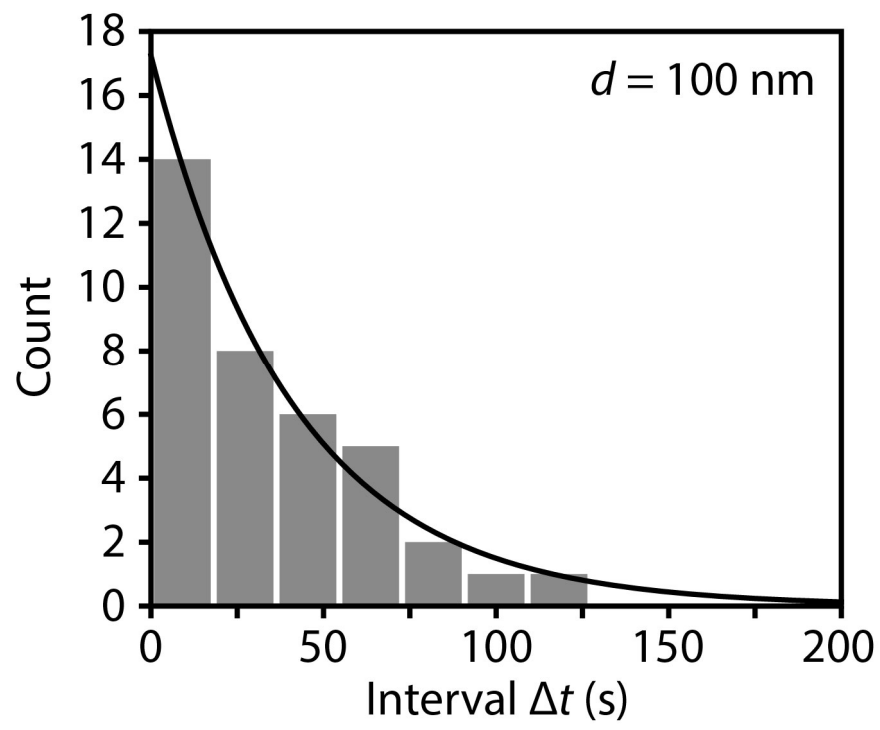

Figure S5. Histogram of the time intervals Δt between successive binding events of PS particles with d = 100 nm. The black line represents an exponential fit.

S7. Concentration dependence of the event rate

We performed additional measurements at different particle concentrations and quantified the corresponding event rates over a measurement duration of 60 min. The results are shown in Figure S6. After sample introduction, all measurements were performed under identical quiescent conditions without external flow. The temperature and illuminated area were kept constant throughout the measurements, while only the particle concentration was varied. Under these controlled conditions, the event rate was found to scale approximately linearly with particle concentration. This concentration dependence is consistent with independent stochastic adsorption events and provides additional evidence that the observed discrete wavelength steps originate from individual particle-binding events rather than instrumental artifacts or random baseline fluctuations.

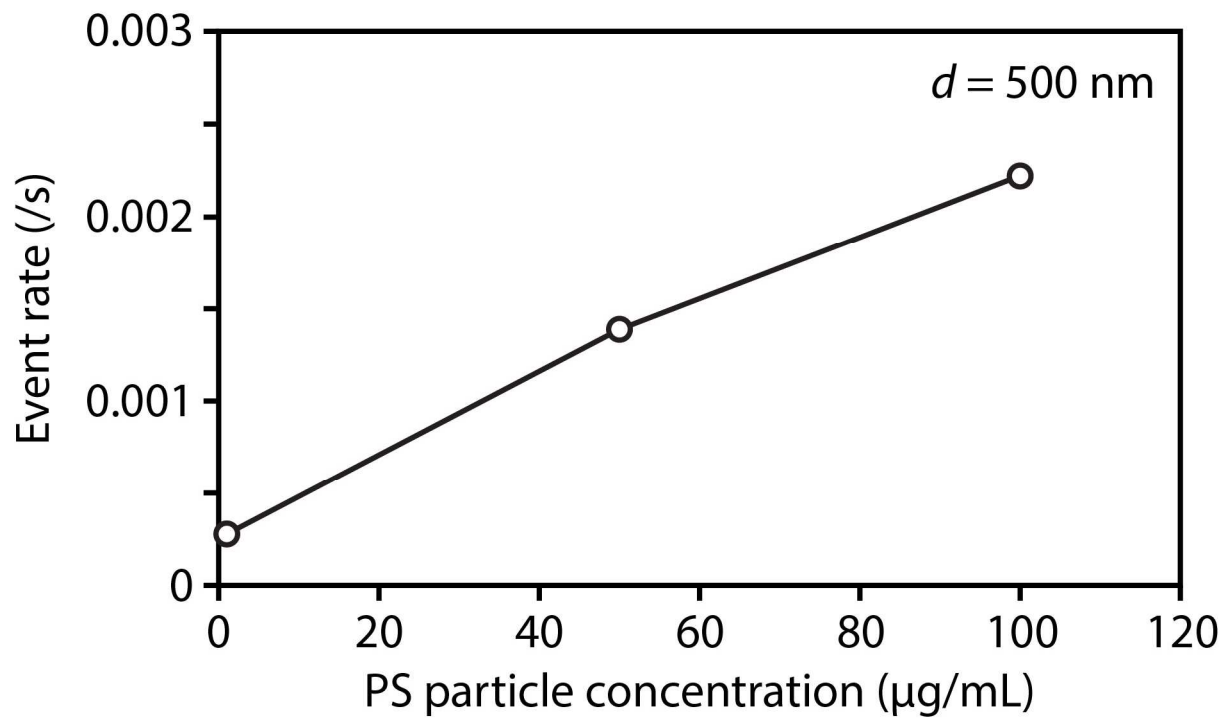

Figure S6. Event rate as a function of PS particle (d = 500 nm) concentration.

S8. Evaluation of the wavelength drift rate

In some measurements, we observed a gradual baseline wavelength drift, which is likely caused by thermally induced refractive index changes in the solution. To quantify the long-term baseline drift of Figure 5, we analyzed the intervals between successive binding events identified by the change-point detection algorithm. For each interval, data points within ±10 frames of the detected binding events were excluded, and the remaining data were linearly fitted. All the extracted wavelength drifts are shown in Figure S7. The drift rates exhibited both positive and negative values, and the average drift rate was found to be 0.00035 pm/s, which is close to zero. Although the cumulative drift over the entire 3000 s measurement corresponds to 1.06 pm, the drift during the typical interval between successive binding events (244 s) was only 0.086 pm. This value is much smaller than the average step height of 1.68 pm for the 500 nm particles, indicating that the step-like wavelength changes remain resolvable.

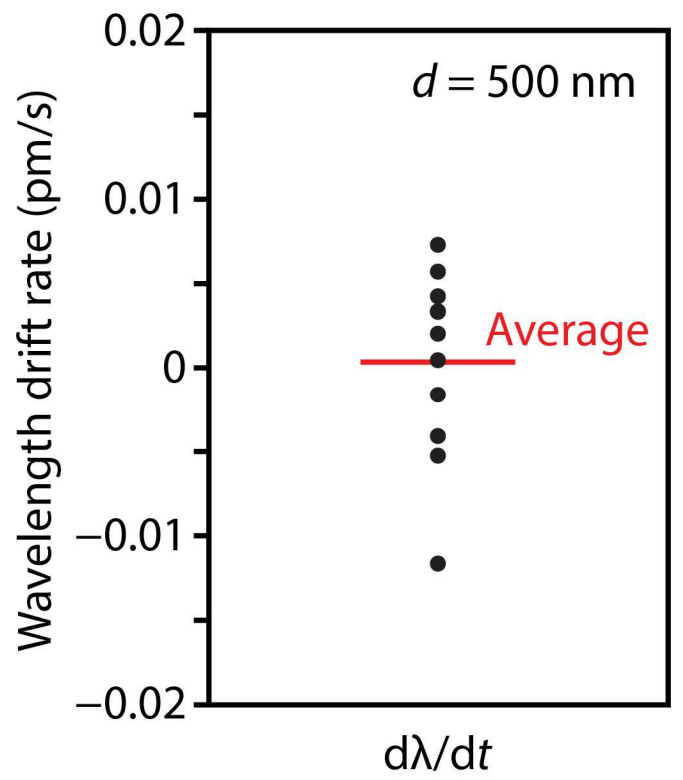

Figure S7. Wavelength drift rates extracted from the intervals between successive binding events of 500 nm PS particles during the 3000 s measurement.
